# How to prevent missing data in cost-effectiveness analyses alongside cardiovascular disease trials? A review on methods of administration of patient-reported outcomes

**DOI:** 10.1186/s41687-026-01108-y

**Published:** 2026-06-30

**Authors:** Manon I. Generaal, Emilien C. J. Wegerif, Çağdaş Ünlü, Gert J. de Borst, Miriam P. van der Meulen

**Affiliations:** 1 University Medical Center Utrecht, Utrecht, Netherlands; 2 Noordwest Ziekenhuisgroep, Alkmaar, Netherlands; 3 Reinier de Graaf Hospital, Delft, Netherlands

**Keywords:** Patient-reported outcomes, Non-response, Cardiovascular disease, Missing data

## Abstract

**Aim:**

Missing data in patient-reported outcomes (PROs) for cost-effectiveness analyses is a widespread challenge that could bias the outcome. This study aims to determine whether methods of administering PROs are associated with missing data and to identify which factors contribute to missing data.

**Methods:**

A systematic literature search was conducted using the terms “cardiovascular disease”, “cost”, and “economic evaluation”. Only cost-effectiveness analyses alongside clinical trials that use PROs to assess quality of life were included. The method of administration of PROs, proportion of missing data, how missing data was handled statistically, and other study characteristics were extracted. Proportions of missing data per administration strategy were calculated.

**Results:**

In total, 123 articles were included in this review, median sample size was 441 (range 50 − 29,759). Median rate of missing data was 13% (range 0-44.2%) but was not reported in 35 (28.7%) studies. How PROs were administered was not reported in 36 (29.3%) studies. A combination of methods to administer PROs resulted in the least missing data (10.4%). Postal questionnaires alone had the most missing data (24.0%), while adding telephone reminders to fill in the questionnaire cut this nearly in half (12.6%). Most studies administered PROs during visits, resulting in 18.0% missing data. How missing data were addressed statistically was not reported in 32 (26.0%) studies.

**Conclusions:**

Reporting on administration of PROs, amount of missing data, and statistical handling of missing data was often lacking. The use of a reminder or a combination of methods to administer PROs seems the most effective approach to substantially decrease the proportion of missing data.

**Clinical trial number:**

Not applicable.

## Introduction

Non-response is a common issue in patient-reported outcomes (PROs). In randomized controlled trials (RCTs), only 75.0% (range 26.0–99.2%) of PRO data are generally available at the primary analysis time point. Only 40% of studies report the number of participants with available data [1]. Even though PROs are an essential part of the cost-effectiveness data, missing data also affects cost-effectiveness analyses (CEAs). One review found 60–85% completeness, reported in only 30 of 88 studies [2]. Another showed a median of 63% complete data [3], and a third reported around 36% missing cost and quality-of-life data, though in both reviews, this was calculable in less than half the studies [4]. 

Missing PRO data can reduce the power of a study, introduce potential bias in outcomes, undermine the obtained effects of randomization by potentially affecting the comparability of the randomized groups, and complicate the statistical handling of data [5]. Every statistical method has to make assumptions about the mechanism of missing data: missing completely at random (MCAR), missing at random (MAR), or missing not at random (MNAR). With MNAR, the missingness is related to the unobserved, missing data (for example, when people with worse outcomes are less likely to respond). When MNAR occurs, imputing missing data can bias results, as the reasons for non-response are linked to the outcome of interest. Among six studies comparing responders and non‑responders, where non‑responders who failed to fill in the questionnaire were contacted by telephone or email to obtain PRO data, two studies reported worse or trending worse outcomes, one reported better outcomes, and three found no statistically significant differences [6]. 

If data is MCAR or MAR, imputation can be used. Complete case analysis and multiple imputation were most commonly used to handle missing data in CEAs [2–4], but both methods could affect CEA outcomes, especially when over 20% of data is missing [7–9]. While a lot of research focuses on how to handle missing data in CEAs [10–14], very little research could be found on how to improve response rates and thus prevent missing data beforehand in CEAs.

To improve response rates of PROs for clinical outcomes, a Cochrane review found that monetary incentives, telephone reminders, and placing questions on clinical outcomes such as adverse events last instead of first (i.e. after PROs) doubled the odds of response. However, this Cochrane review did not focus on CEAs, recent trials, or on the methods of admistration [15]. The current review aimed to assess whether the method of PRO administration (in-hospital, telephone, or mail) influenced missing data in CEAs conducted alongside cardiovascular trials.

## Methods

This review aimed to answer three questions: (I) which strategies were used to administer PROs in cost-effectiveness analyses alongside cardiovascular disease (CVD) trials, (II) how these strategies impact the amount of missing PRO data, and (III) what other aspects are associated with the amount of missing data, such as sample size, or illness category. Cardiovascular disease was chosen as the focus of this review to ensure feasibility, and because PRO use and data completeness may differ across disease areas. In addition, cardiovascular disease is a field in which PROs are frequently used.

The PRISMA-ScR checklist was used for reporting in this review [16]. The protocol of this review was registered in PROSPERO: CRD42024529920.

### Search

The index terms “cardiovascular disease”, “cost”, and “economic evaluation” were applied. All synonyms and/or closely related words of these index terms were added, and the definitive search was compiled by a medical database specialist. The databases of PubMed, Embase, and Web of Science were searched on 24-04-2024. The search term for PubMed can be found in Table A1 (Appendix).

### Inclusion and exclusion criteria

Articles were included if they reported cost-effectiveness analyses of a CVD trial using PROs, or when individual patient-level data from PROs from another trial were used. The focus of PROs in this review was quality-of-life questionnaires that were administered during a trial and used in the CEA. Studies that used multiple different sources of PROs, reviews, expert opinions, case reports, articles published before 2000, articles published in a language other than English, and articles where full text could not be retrieved were excluded.

### Data extraction

Title and abstract screening was executed by two independent researchers (MG and EW) via Rayyan [17]. When finished, the screening results were unblinded and checked for differences in eligibility. Disagreement (10.2%) was discussed and resolved. Full text screening was done in EndNote [18], which was used to collect all publications. One researcher extracted the data, and the other researcher verified the extracted data. The author, year of publication, country, study period, study design, sample size, mean age, male/female ratio, description of PRO administration, description of which PRO was used for quality of life and costs, description of missing data, use of complete case analysis or imputation, and the utility estimate were extracted. In case of missing items, the protocol or study design paper of the study was sought. Excel [19] was used to collect all the data. R Statistical Software (R version 4.4.1, 2024-06-14) [20] was used to perform the statistical analyses.

### Statistical analysis

To answer the first research question, all extracted data on the method of PRO administration were displayed in a table. In addition, the methods of administration were grouped into the following groups: telephone, post, post with reminder, self-administered, hospital or home visit (hereafter: visit), combination of methods, and not reported. For example, when the method in an article was described as ‘questionnaires were filled in at visits or via telephone’, then it was put in the category ‘combination’.

For the second research question, the proportion of missing PRO data at 6 months (after baseline) for each study was presented in the corresponding group of questionnaire administration strategies. Missing data were defined as entirely missing PRO questionnaires. If 6 months data were not available, the first time point or the one closest to 6 months after baseline was chosen. The mean was calculated and visualized in a bar plot. If the proportion of missing PRO data per time point was not reported, it was assumed that the proportion of missing data of the whole study was similar at each time point. If missing data was not mentioned in the article, the study was excluded from the calculation of the proportion of missing data. To answer the third research question, plots were made of the proportion of missing data and sample size, illness category, and method of missing data handling.

## Results

**Fig. 1:**
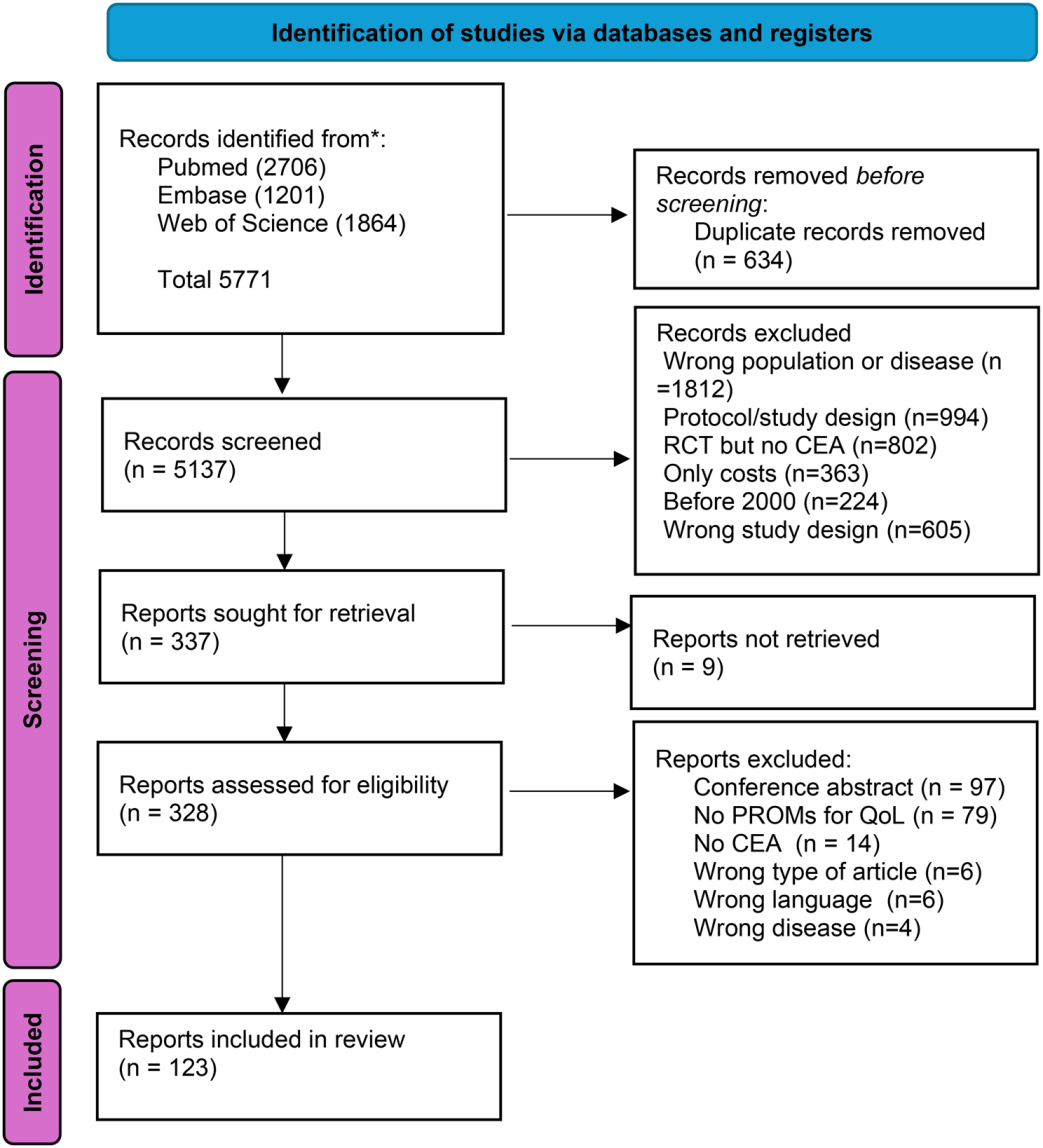
Flow diagram. CEA = cost-effectiveness analysis, PROMS = patient reported outcome measures, RCT = randomized controlled trial, QoL = quality of life

The search resulted in 5771 records, of which 634 records were removed as duplicates (Fig. 1). After screening the title and abstract, 337 records were left for full-text screening. Full text screening resulted in 123 records to be included in the review. The most important reasons for exclusion in this stage were that abstracts were conference abstracts or that the study did not use any PROs.

Most of the reports were in the field of coronary artery disease (61.8%), followed by stroke (22.0%), and peripheral artery disease (15.4%) (see Table 1). The median sample size of the reports was 441 (range 50-29759). The mean age was 65 (SD 6.7) and the median percentage of females in the study was 31.0% (IQR 22–42). The median amount of missing data was 13% (range 0-44.2%), but was not reported in 35 of the 123 (28.7%) included studies. How missing data were handled statistically was not reported in 34 of 123 (27.6%) of studies. When reported, multiple imputation was the most used technique, in 43 of 123 (35.0%) studies, followed by complete case analysis (11.5%), last observation carried forward, and mean imputation (both 5.7%) (see Table 2).

**Table 1:** Overview of included reports

Variable	Number of reports (%) / Mean ± SD
*Illness Category*	
* Coronary artery disease*	76 (61.8%)
* Stroke (ischemic)*	27 (22.0%)
* Peripheral artery disease*	19 (15.4%)
* Combined (all three)*	1 (0.8%)
*Questionnaire administration*	
* Online with reminder*	1 (0.8%)
* Post*	10 (8.1%)
* Post with telephone reminder*	4 (3.3%)
* Self-administered*	15 (12.2%)
* Telephone*	10 (8.1%)
* Visit*	33 (26.8%)
* Combination*	14 (11.4%)
* Not reported*	36 (29.3%)
*Sample size*	Range 50 − 29,759 Median 441 (IQR 182-1,147)
*Age*	65.3 ± 6.7
*Proportion female*	Range 0-77.1% Median 31% (IQR 22–42%)
*Proportion of missing data*	Range 0-44.2% Median 13% (IQR 6–22%)
* Missing data not reported*	35/123 records (28.7%)
*Analysis*	
* Complete case* Imputation* * Not reported*	14 (11.5%) 74 (60.7%) 34 (27.9%)

*See Table 2 for more detailed descriptions of which imputation methods were used

**Table 2:** Overview of methods used to handle missing data

Missing data handling of health utility	Number of reports (%)
*Multiple imputation*	43 (35.2%)
*Not reported*	34 (27.9%)
*Complete case analysis*	14 (11.5%)
*Mean imputation*	8 (6.6%)
*LOCF imputation*	7 (5.7%)
*Imputation*,* not specified*	6 (4.9%)
*Linear regression imputation*	4 (3.3%)
*Best*,* worst*,* median*,* baseline imputation*	1 (0.8%)
*Bootstrap resampling imputation*	1 (0.8%)
*Linear interpolation and extrapolation*	1 (0.8%)
*Mean imputation and LOCF imputation*	1 (0.8%)
*Mode*,* mean*,* fixed value imputation*	1 (0.8%)
*Prediction and LOCF imputation*,* and complete case analysis*	1 (0.8%)

LOCF = last observation carried forward

The method for administering PROs was most often not reported (*N* = 33, 29.3%), after which the visit (*N* = 33, 26.8%) was most often used as method to administer PROs (see Table 2). Postal questionnaires were used in 10 (8.1%) studies, a telephone reminder was added in 4 (3.3%). 14 (11.4%) studies used a combination of methods. Lastly, 15 (12.2%) studies reported that the questionnaire was self-administered, but did not specify which method was used to administer the questionnaires.

**Fig. 2:**
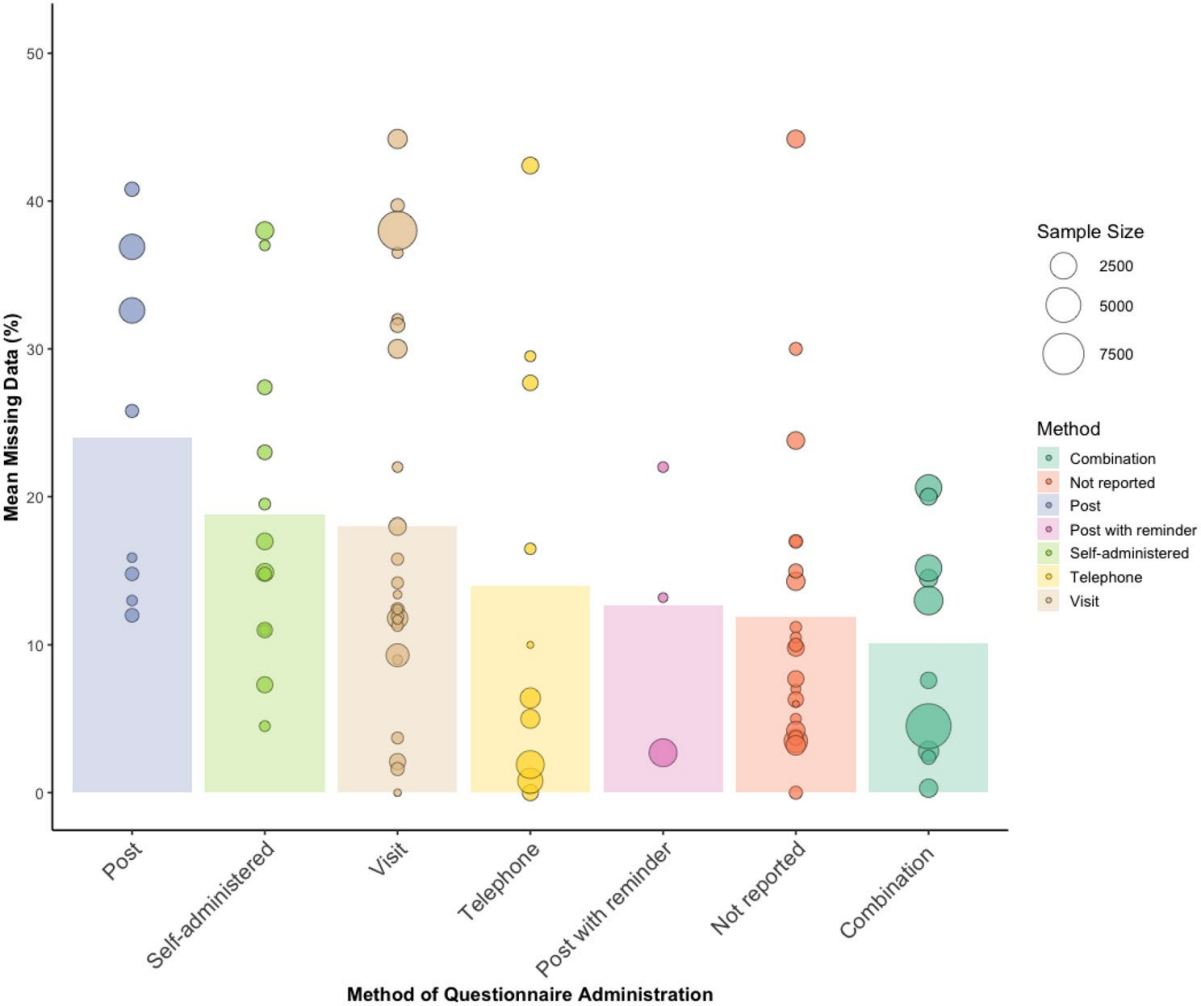
Mean missing data by method of questionnaire administration

The methods of questionnaire administration that resulted in the highest amount of missing data were (I) post, (II) self-administered, and (III) visit with 24.0, 18.8, and 18.0% missing data, respectively (Fig. 2). Sending the questionnaire via post, but adding a telephone reminder resulted in 12.6% missing data. The methods with the least amount of missing data were when a combination of methods was used (10.1% missing data) (See Appendix Table A2). There were 15 studies that reported the method of questionnaire administration, but not the proportion of missing data.

**Fig. 3:**
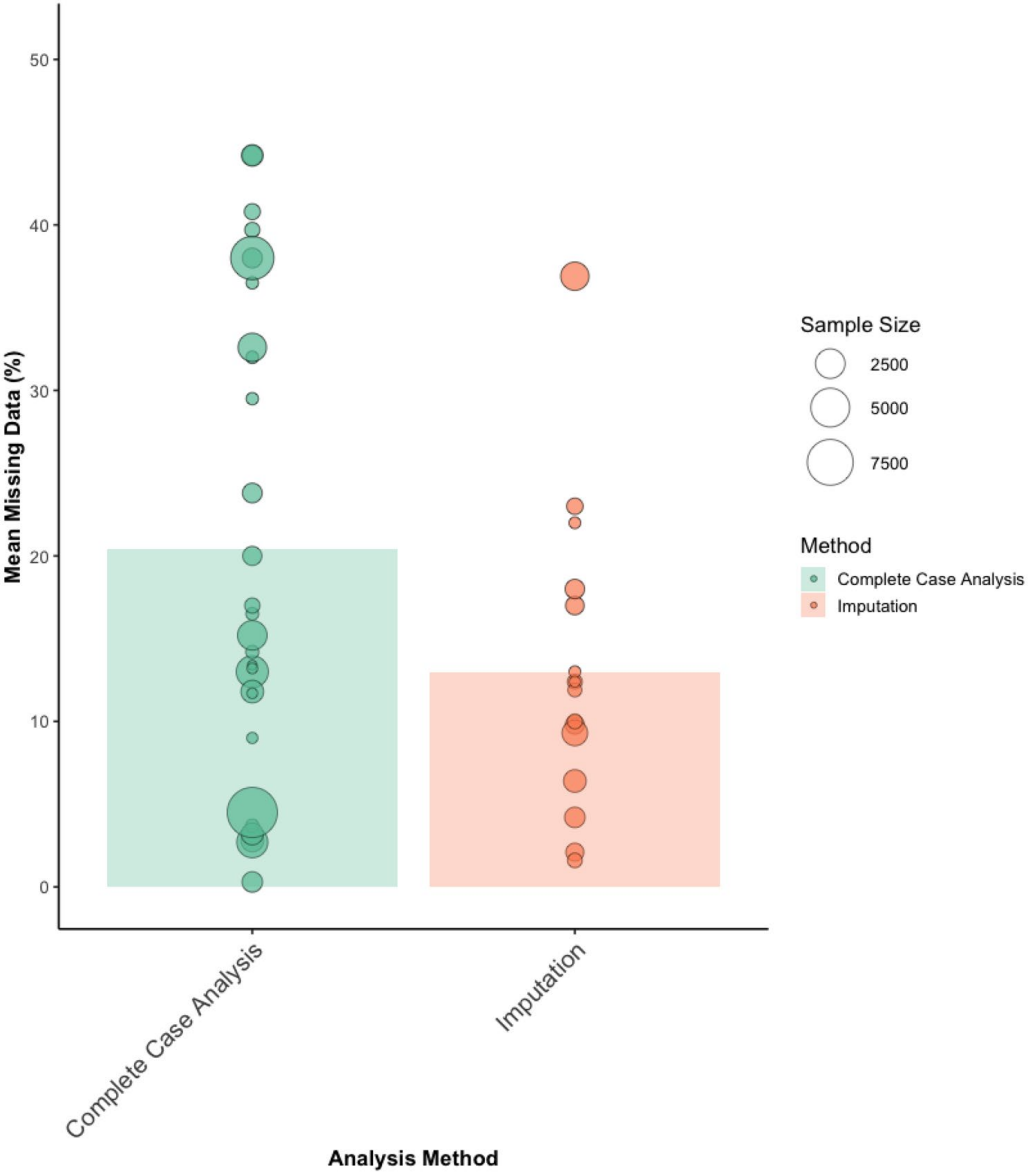
Missing data by analysis method

**Fig. 4:**
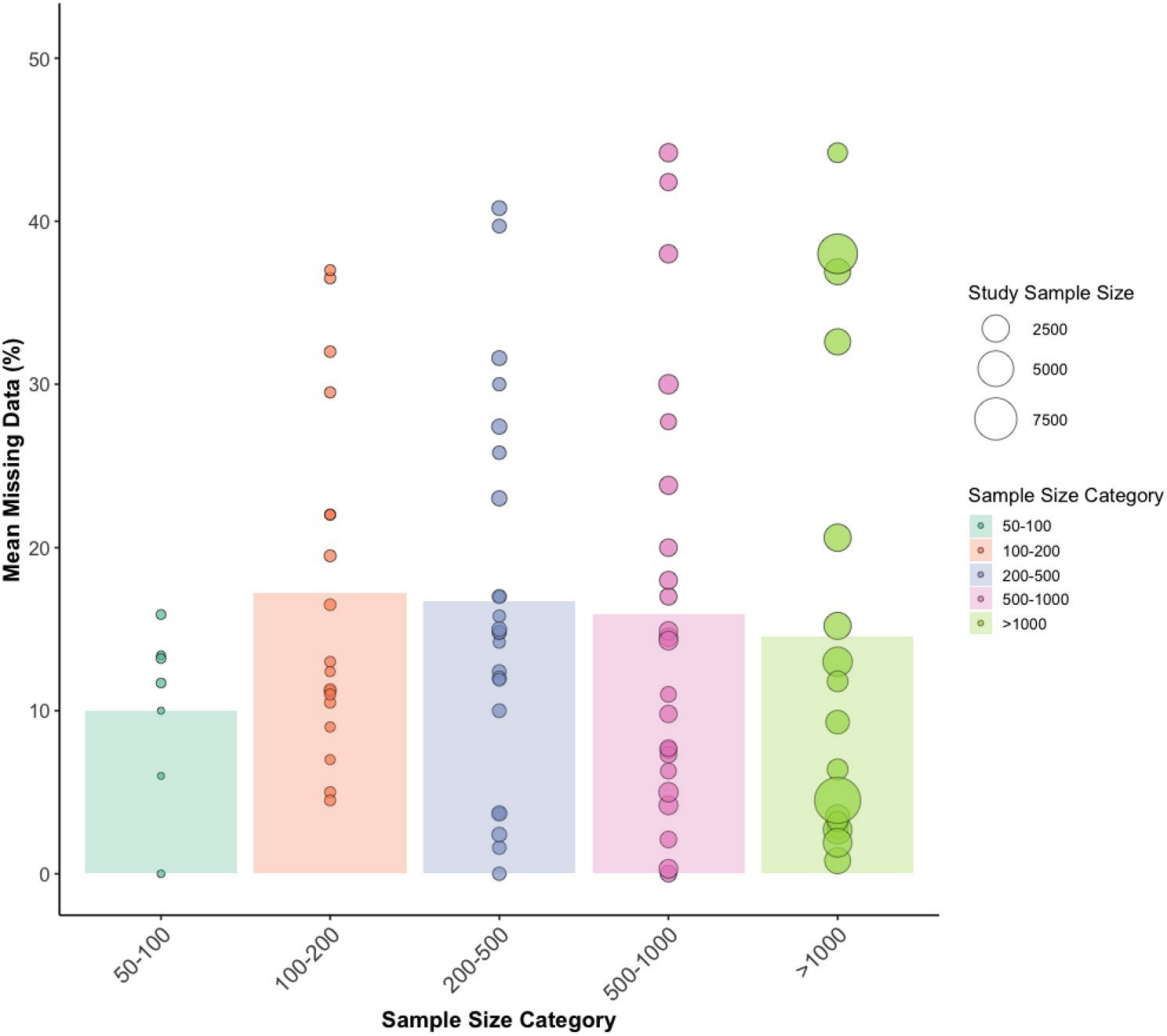
Missing data per sample size

**Fig. A1:**
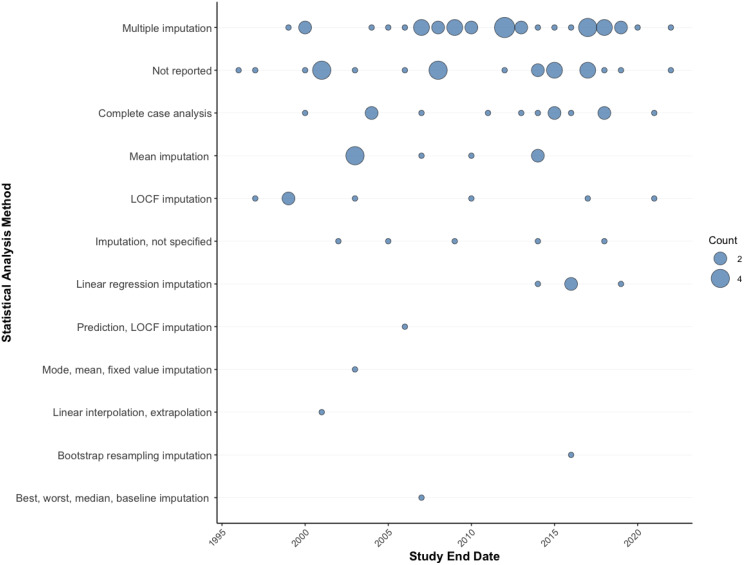
Statistical analysis method over time

**Fig. A2:**
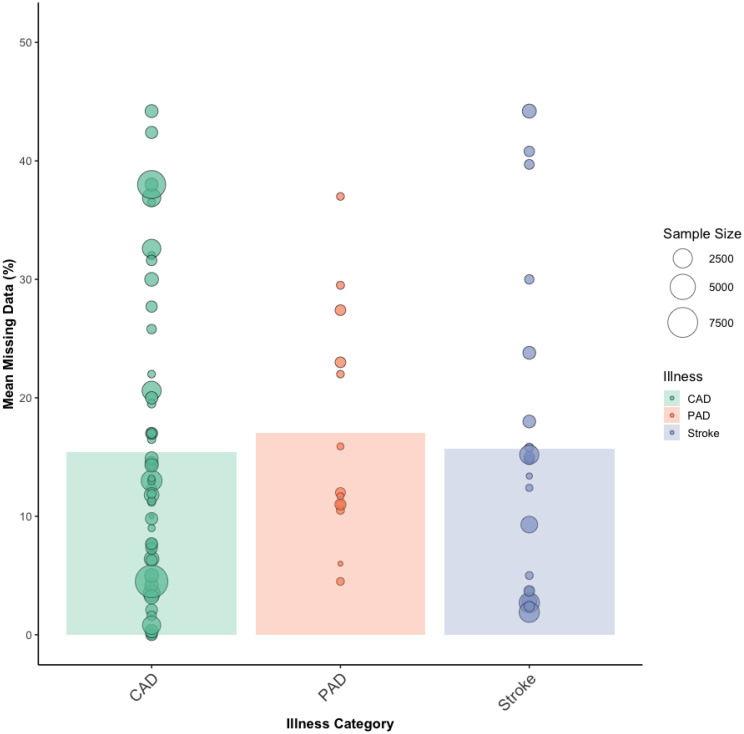
Missing data per illness category

**Fig. A3:**
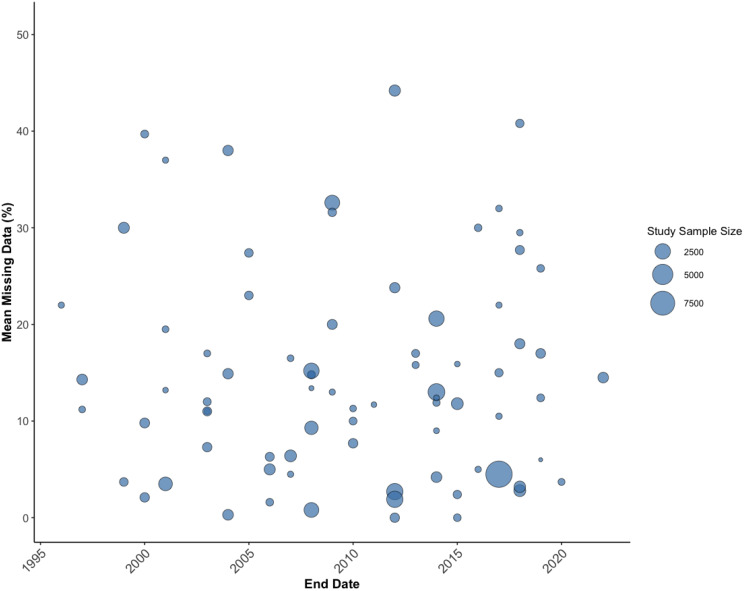
Mean missing data over time

The proportion of missing data was higher in the studies that used complete case analysis than in those that used imputation methods (See Fig. 3). There was less missing data when the sample size was smaller, between 50 and 100 (see Fig. 4). The statistical methods used to handle missing data do not seem to change over time (see Appendix Figure A1). The proportion of missing data was similar for coronary artery disease, peripheral artery disease, and stroke (see Appendix Figure A2). There was no clear temporal trend in the amount of missing data over time (see Appendix Figure A3).

## Discussion

This review examined which methods were used to administer patient-reported outcomes in cost-effectiveness analyses alongside cardiovascular trials, how these methods impacted the proportion of missing data, and whether other factors were associated with missing data. It showed that the method of administration of PROs, the amount of missing data, and the statistical handling of missing data was often lacking. The use of a telephone reminder to fill in the questionnaire or a combination of methods to administer PROs seems to be the most effective approach to decrease the proportion of missing data, while a postal questionnaire alone is associated with the highest proportion of missing data.

The median of missing data in studies (13%) is lower in this review compared to previous reviews on CEAs alongside trials, which reported 15–40% missing data [2–4]. This could suggest that more recent studies had less missing data, but we could not confirm this. It could also be explained by our focus on cardiovascular trials, which was not the specific focus of the other reviews. Reporting of missing data was also somewhat better: one-third of studies lacked this information, versus over half in earlier reviews. However, details on questionnaire administration and how missing data were handled were still missing in about one in four studies. Overall, reporting in CEAs alongside trials in cardiovascular patients remained inadequate.

A possible explanation is that the CHEERS checklist, which is commonly used in economic evaluations, does not explicitly recommend reporting missing data [21]. In contrast, the CONSORT-PRO extension, which guides PRO reporting in RCTs, does recommend it [22]. Adding missing data reporting to CHEERS could improve transparency. Given that the handling of missing data (e.g., complete case analysis, imputation methods) can influence cost-effectiveness results, this information is essential for evaluating study quality [8, 9, 23]. Elements on missing data that could be added to the CHEERS are a description of the missing data, the method used to handle missing data, and limitations of the missing data or the handling. More detailed recommendations on the reporting of missing data are available in literature [24]. 

Most studies used the hospital or home visit as an opportunity to administer the PROs, however, the mean proportion of missing data in these studies was relatively high. The optimal strategy, in terms of response, to administer PROs was a combination of strategies. Interestingly, the ISPOR PRO Mixed Modes Good Research Practices Task Force advises against mixing modes to administer questionnaires to avoid adding measurement error into the trial [25]. The discussion would then be to either have a lower amount of missing data by using a combination of methods in your trial, but increase the chance of measurement error bias, or less measurement error but more missing data. The second-best strategy to administer questionnaires was sending a postal questionnaire with a telephone reminder. This is similar to one of the outcomes of the Cochrane review, where adding a reminder to postal questionnaires doubled the odds of response [15]. For both these strategies, the interpretation of why response is higher could be twofold: combining methods could work better as patients could have different preferences, or the effect could be due to the reminders that are sent.

The optimal methods to collect PROs in CEAs alongside trials that were found in this review are strategies that will result in a high workload for the research team. As CEAs are often a secondary outcome of a clinical trial, a high workload only for the CEA will generally be unachievable or too costly. New methods should be studied in which PRO-response to different methods of administration is compared directly, to assist selection of an appropriate method with a maximized response and/or a minimized workload.

Lastly, many studies included in this review did not impute missing data or used a suboptimal imputation method to address the missing data. These methods can influence the outcome and uncertainty of the cost-effectiveness analysis, consequently impacting the probability of an intervention being cost-effective or not [23]. Future cost-effectiveness analyses alongside trials are recommended to follow guidance on how to handle missing data outlined in previous literature [11, 24]. In addition, the limited information on how missing data was imputed and the finding that suboptimal imputation methods were used underscores the need to prevent missing PRO data, to limit bias introduced by use of suboptimal imputation methods.

Strengths of this review are the identification of strategies that should lead to a lower amount of missing data when designing a cost-effectiveness analysis alongside a clinical trial. In addition, our review shows that reporting on missing data or on strategies to prevent or cope with missing data in these analyses could still be improved. This study was limited to patients with cardiovascular disease, which may affect generalizability to other patient groups. Another limitation is that due to the poor reporting in studies, the current study had to deal with a high amount of ‘missing data’. Data that were not reported in the studies could not be included in the analyses on the proportion of missing data per PRO-administration strategy, illness category, sample size, etc. As is customary in review, we only used the available information in the study analysis in this review, thus exactly performing a complete case analysis ourselves. As we could not justify whether the assumption that our own data was *not* MNAR (e.g., high proportion of missings in studies may not be reported, this would be the most appropriate choice. Another consideration is that this review is descriptive; thus, it is not possible to infer causality between the administration strategy of the PRO used and the proportion of missing data. Nonetheless, the results of this review show the variation in missingness observed across the administration strategies. While this variation was high within the administration categories, in the category of studies that used a combination of methods or a postal reminder, there was no high outlier (in contrast to the other categories). However, in the category of postal reminders, this could also be due to the small number of studies (three).

Underreporting of missing data not only raises methodological concerns, as outlined earlier, but also has ethical concerns if it hinders the usefulness or incorrect interpretation of available data. The lack of transparency on missing data can lead to misleading conclusions and reduced generalizability of results. In addition, it could mask important patterns when missing data affects certain (vulnerable) groups. When the results of these studies are used in policy decisions, it might lead to inequality as certain groups were (unknowingly) overlooked. It is therefore crucial to enhance transparency on the amount of missing data and methods used to guide interpretation of results, for instance by including these aspects in the CHEERS checklist. In addition, further research should be performed to optimize methods of administration.

## Conclusions

Reporting on the administration of PROs, the amount of missing data, and the statistical handling of missing data was often lacking. For trial-based cost-effectiveness analyses, using a telephone reminder to fill in the questionnaire or a combination of methods to administer PROs seems to be the most effective approach to decrease the proportion of missing data substantially.

## Data Availability

The data that support the findings of this study are available from the corresponding author upon reasonable request.

## Appendix

**Table A1:** PubMed search

Search number	Query
30	(#1 OR #2 OR #3 OR #4 OR #5 OR #6 OR #7 OR #8 OR #9 OR #10 OR #14 OR #20 OR #23 OR #27) AND (((“randomized“[Title] OR “randomised“[Title] OR “trial“[Title]) AND (“economic“[Title/Abstract] OR “cost*“[Title/Abstract])) NOT (“pilot“[Title] OR “feasibility“[Title]))
29	((“randomized“[Title] OR “randomised“[Title] OR “trial“[Title]) AND (“economic“[Title/Abstract] OR “cost*“[Title/Abstract])) NOT (“pilot“[Title] OR “feasibility“[Title])
27	(“peripheral arterial disease“[MeSH Terms] OR “peripheral arterial disease*“[Title/Abstract] OR “peripheral artery disease*“[Title/Abstract] OR “lower extremity arterial disease*“[Title/Abstract] OR “lower extremity artery disease*“[Title/Abstract] OR “chronic limb ischemia“[Title/Abstract] OR “chronic limb threatening ischemia“[Title/Abstract] OR “intermittent claudication“[Title/Abstract])
24	#1 OR #2 OR #3 OR #4 OR #5 OR #6 OR #7 OR #8 OR #9 OR #10 OR #14 OR #20 OR #23
23	#21 NOT #22
22	intracranial hemorrhage, traumatic[majr]
21	intracranial hemorrhages[majr]
20	#15 AND (#16 OR #17 OR #18 OR #19)
19	brainstem[tiab]
18	cerebral[tiab]
17	cerebrovascular[tiab]
16	brain[tiab]
15	stroke[tiab]
14	#11 AND (#12 OR #13)
13	disease[tiab]
12	artery[tiab]
11	coronary[tiab]
10	cardiovascular mortality[tiab]
9	heart diseases[majr: noexp]
8	death, sudden, cardiac[majr]
7	cerebrovascular accident[tiab]
6	brain ischemia[majr: noexp]
5	stroke[majr]
4	coronary disease[majr]
3	myocardial infarction[majr]
2	cardiovascular diseases[majr: noexp]
1	cardiovascular disease*[tiab]

**Table A2:** Description of ‘combination’ of methods

Source	Method of questionnaire adminstration
^1^	Telephone and e-mail
^2^	Clinical follow-up or telephone
^3^	Telephone, then postal questionnaire, or face-to-face if patient is in hospital
^4^	Post or online
^5^	Visit or telephone
^6^	Telephone or post
^7^	Baseline and 12 months face-to-face, 6 month postal or electronic questionnaire
^8^	3 and 12 months telephone, 6 months home visit
^9^	Mail at 3 months + telephone reminder, at visit during baseline and 12 months
^10^	Postal or telephone

### References Appendix

1. Aarts GWA, Camaro C, Adang EMM, et al. Pre-hospital rule-out of non-ST-segment elevation acute coronary syndrome by a single troponin: final one-year outcomes of the ARTICA randomised trial. *European Heart Journal-Quality of Care and Clinical Outcomes* 2024. DOI: doi:10.1093/ehjqcco/qcae004.
2. Baron SJ, Wang K, House JA, et al. Cost-Effectiveness of Transcatheter Versus Surgical Aortic Valve Replacement in Patients With Severe Aortic Stenosis at Intermediate Risk Results From the PARTNER 2 Trial. *Circulation* 2019; 139: 877–888. DOI: doi:10.1161/circulationaha.118.035236.
3. Bhattarai N, Price CI, McMeekin P, et al. Cost-effectiveness of an enhanced Paramedic Acute Stroke Treatment Assessment (PASTA) during emergency stroke care: Economic results from a pragmatic cluster randomized trial. *Int J Stroke* 2022; 17: 282–290. DOI: doi:10.1177/17474930211006302.
4. Dixon P, Hollinghurst S, Edwards L, et al. Cost-effectiveness of telehealth for patients with raised cardiovascular disease risk: evidence from the Healthlines randomised controlled trial. *BMJ Open* 2016; 6: e012352. DOI: doi:10.1136/bmjopen-2016-012352.
5. Filipovic-Pierucci A, Dur, -Zaleski I, et al. Polymer-free drug-coated coronary stents are cost-effective in patients at high bleeding risk: Economic evaluation of the LEADERS FREE trial. *EuroIntervention* 2018; 13: 1688–1695. DOI: doi:10.4244/EIJ-D-17-00286.
6. Stokes EA, Lazaroo MJ, Clout M, et al. Cost-effectiveness of the i-gel supraglottic airway device compared to tracheal intubation during out-of-hospital cardiac arrest: Findings from the AIRWAYS-2 randomised controlled trial. *Resuscitation* 2021; 167: 1–9. DOI: doi:10.1016/j.resuscitation.2021.06.002.
7. Te Ao B, Harwood M, Fu V, et al. Economic analysis of the ‘Take Charge’ intervention for people following stroke: Results from a randomised trial. *Clin Rehabil* 2022; 36: 240–250. DOI: doi:10.1177/02692155211040727.
8. Verweij L, Petri ACM, MacNeil-Vroomen JL, et al. The Cardiac Care Bridge transitional care program for the management of older high-risk cardiac patients: An economic evaluation alongside a randomized controlled trial. *PLoS One* 2022; 17: e0263130. DOI: doi:10.1371/journal.pone.0263130.
9. Wagner TH, Hattler B, Bishawi M, et al. On-pump versus off-pump coronary artery bypass surgery: cost-effectiveness analysis alongside a multisite trial. *Ann Thorac Surg* 2013; 96: 770–777. DOI: doi:10.1016/j.athoracsur.2013.04.074.
10. Effect of intermittent pneumatic compression on disability, living circumstances, quality of life, and hospital costs after stroke: secondary analyses from CLOTS 3, a randomised trial. *Lancet Neurol* 2014; 13: 1186–1192. DOI: doi:10.1016/s1474-4422(14)70258-3.

## Abbreviations

CEAs: Cost-effectiveness analyses
CVD: Cardiovascular disease
LOCF: Last observation carried forward
PROs: Patient-reported outcomes (PROs)
RCTs: Randomized controlled trials
MAR: Missing at random
MCAR: Missing completely at random
MNAR: Missing not at random
